# Improving assessment of child growth in a pediatric hospital setting

**DOI:** 10.1186/s12887-020-02289-1

**Published:** 2020-09-03

**Authors:** Priya M. Gupta, Emily Wieck, Joel Conkle, Kristina A. Betters, Anthony Cooley, Selena Yamasaki, Natasha Laibhen-Parkes, Parminder S. Suchdev

**Affiliations:** 1 Nutrition and Health Sciences Program, Laney Graduate School, Emory University, Atlanta, GA USA; 2 Department of Pediatrics, Emory University School of Medicine, Atlanta, GA USA; 3 Health and Nutrition Section, UNICEF, Windhoek, Namibia; 4 Department of Pediatrics, Vanderbilt University, Nashville, TN USA; 5 Children’s Healthcare of Atlanta, Atlanta, GA USA

**Keywords:** Child health, Growth, Anthropometry, Pediatrics

## Abstract

**Background:**

Accurate anthropometric measurements are essential for assessing nutritional status, monitoring child growth, and informing clinical care. We aimed to improve height measurements of hospitalized pediatrics patients through implementation of gold standard measurement techniques.

**Methods:**

A quality improvement project implemented computerized training modules on anthropometry and standardized wooden boards for height measurements in a tertiary children’s hospital. Heights were collected pre- and post-intervention on general pediatric inpatients under 5 years of age. Accuracy of height measurements was determined by analyzing the variance and by comparing to World Health Organization’s defined biologically plausible height-for-age z-scores. Qualitative interviews assessed staff attitudes.

**Results:**

Ninety-six hospital staff completed the anthropometry training. Data were available on 632 children pre- and 933 post-intervention. Training did not increase the proportion of patients measured for height (78.6% pre-intervention vs. 75.8% post-intervention, *p* = 0.19). Post-intervention, wooden height boards were used to measure height of 34.8% patients, while tape measures and wingspan accounted for 42.0% and 3.5% of measurements, respectively. There was no improvement in the quality of height measurements based on plausibility (approximately 3% height-for-age z-scores measurements flagged out of range pre- and post-intervention), digit preference (13.4% of digits pre- and 12.3% post-intervention requiring reclassification), or dispersion of measurements (height-for-age z-scores standard deviation 1.9 pre- and post-intervention). Staff reported that using the wooden board was too labor consuming and cumbersome.

**Conclusions:**

Our findings suggest that efforts to improve anthropometric measurements of hospitalized children have multiple obstacles, and further investigation of less cumbersome methods of measurements may be warranted.

## Background

Malnutrition, defined as either protein-energy malnutrition, deficiency in micronutrients, or overnutrition/obesity, is a major cause of childhood morbidity and mortality globally; pediatricians play a vital role in assessing and managing malnutrition [1, 2]. Anthropometry, or the measurement of body parameters, is used clinically to diagnose malnutrition as well as monitor child growth in populations [3, 4]. Routinely collected anthropometric measurements in children include weight, height or length, and head circumference. Obtained measurements are then compared to a reference population using the following sex-specific indices: weight-for-age (underweight), height-for-age (stunting), and weight-for-height (wasting) [5, 6]. The World Health Organization (WHO) and other international organizations have implemented standardized procedures for measuring and interpreting anthropometry [7, 8]. Height can be assessed with numerous methods including measuring wingspan to estimate height, or directly measuring height with a tape measure or stadiometer. It is well documented that using a tape measure to measure height or using wingspan to estimate height yields inaccurate measurements as compared to the gold standard wooden height board [7].

The Academy of Nutrition and Dietetics and the American Society for Parenteral and Enteral Nutrition have called for improvement in identification of pediatric malnutrition [2]. However, screening to detect malnutrition is not routinely implemented, and data quality is often poor [9]. A recent multicenter randomized trial in primary care practices within the United States found that only 30% of height measurements were accurate (defined as within 0.5 cm (cm) of a trained anthropometrist) [10]. Accurate anthropometric measurements are particularly important in inpatient settings for appropriate pharmaceutical dosing that uses weight or total body surface area, as well as early detection of malnutrition which can affect hospital outcomes and duration of stay. Pediatric patients presenting with malnutrition have a higher rate of complications, higher rate of mortality, longer hospital course, and increased hospital costs [2, 11]. Studies in outpatient settings have demonstrated the importance of training and assessment of external reliability to improve the quality of anthropometric measurements [3, 12]. To address barriers in nutritional assessment in hospitalized children, we implemented a quality improvement project to improve measurement of anthropometry among hospitalized children through training and use of standardized equipment.

## Methods

The quality improvement project was conducted at a tertiary care pediatric hospital. Data were abstracted from the electronic medical record (EMR) from 3 units at the Children’s Healthcare of Atlanta in Atlanta, Georgia. Pre-intervention data were collected October 2012 to February 2013, and post-intervention data were collected March 2013 to November 2013. The Children’s Healthcare of Atlanta’s institutional review board classified this quality improvement project as non-human subjects research.

Eligible participants included all patients admitted to the general medicine floors under the age of 5 years regardless of whether a height was measured. Patients who were transferred from the Pediatric Intensive Care Unit, those with a physical disability that prevented accurate measurement (e.g., scoliosis), or other clinical instability at the time of admission were excluded.

The intervention included a computer-based training module on standard anthropometry and dissemination of standard measurement tools including a wooden height board (ShorrBoard®, Weigh and Measure LLC, Maryland USA) and a digital scale (Rice Lake Weighing Systems, Inc., Rice Lake, WI). The module included a brief 15-min instructional program on ensuring accurate height/length measurements using two trained anthropometrists and standardized procedures [7]. All nursing staff, patient care specialists and technicians, and clinical educators on the three included inpatient floors received the training (*n* = 96). Completion of the module was verified by the floor nursing manager. The training module was available for staff completion from 2/1/2013 through 2/15/2013, and wooden height boards were provided to the floors following training completion. Existing digital scales on the floors were used pre- and post-intervention to measure weight.

**Fig. 1:**
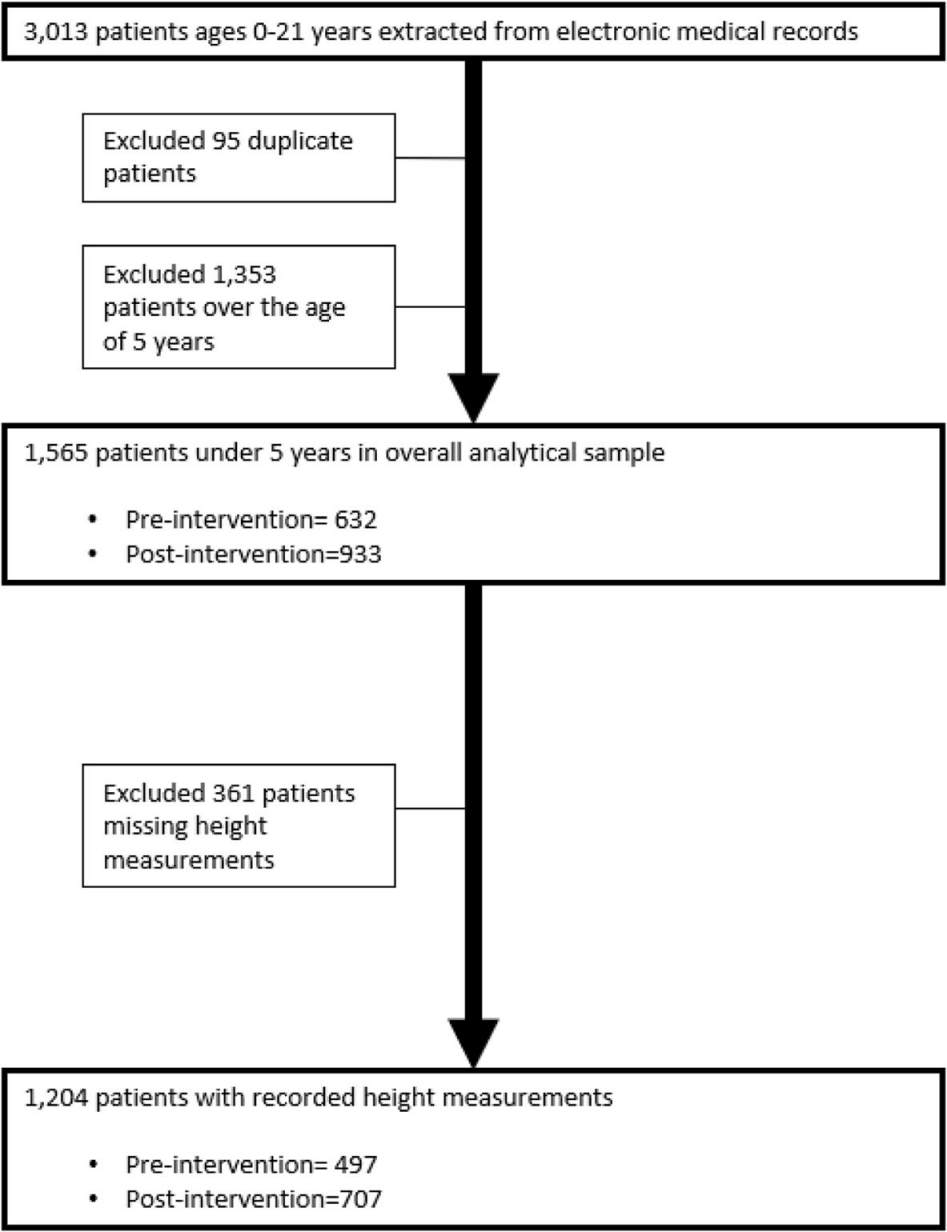
Flowchart of included subjects

Height was measured standing upright for children 2–5 years of age, while length was measured lying down for children under 2 years of age. For the purposes of this paper, the term “height” is used to describe height or length. Height measurements recorded in cm and weight measurements recorded in kilogram (kg) were entered into the EMR per routine protocol. Staff who participated in the intervention were asked to complete a questionnaire on their opinions and reflections of using the standardized equipment for height measurement. Data on 1565 children 0–5 years, 632 pre-intervention and 933 post-intervention, were available for analysis (Fig. 1). Height was measured among 1204 children, of which, 497 were collected pre-intervention and 707 were collected post-intervention.

The WHO Growth Standards [5] were used to calculate z-scores for height-for-age (HAZ), weight-for age (WAZ), and weight-for-height (WHZ) which were compared pre- and post-intervention. Cutoffs of HAZ < -2, WAZ < -2, and WHZ < -2 were used to define stunting, underweight, and wasting, respectively. Z-score plausibility was determined using WHO cutoffs. We used the following WHO-defined standard deviation (SD) ranges to assess the quality of data (HAZ 1.1–1.3, WAZ 1.0–1.2, and WHZ 0.85–1.1) [13, 14]. Flagged values were defined as HAZ < -6 or > 6 SD; WAZ < − 6 or > 5 SD; WHZ < -5 or > 5 SD. We also examined flags used in Demographic and Health Surveys (DHS) for height plausibility (lying down 45–110 cm, standing up 65–120 cm). Levene’s test for equality of variances was used to determine if variances were equal before and after the training.

Digit preference, or human tendency to round to specific decimal numbers more often than others, was assessed for height measurements with each of the possible digits (0–9) in the tenths place (mm). The difference between the expected and observed percentages was calculated and tested for preference of each individual digit using a two-sided binomial test. We used cutoffs of 25% to indicate moderate digit preference [15]. To determine how close observed proportions were to a uniform distribution, we summed the difference between expected and observed for positive differences only to determine the number of observations that would need to be reclassified to achieve a uniform distribution [16].

All analyses were performed using SPSS version 20 (IBM Corp., Armonk, NY, USA). Statistical tests were two-sided and evaluated using an alpha level equal to 0.05. Pearson’s Chi-Square Tests were used to evaluate differences by intervention status.

## Results

Baseline characteristics of enrolled patients pre- and post-intervention are summarized in Table 1. There were no statistically significant differences in the age, sex, race, length of stay, admission floor, nor insurance of patients pre- and post-intervention (*p* > 0.05). Diagnoses at admission included viral upper respiratory infections, asthma exacerbations, and soft tissue infections such as cellulitis and abscesses (data not shown). Some patients also had chronic underlying conditions including cystic fibrosis, diabetes mellitus, muscular dystrophy, osteogenesis imperfecta, sickle cell disease, and ulcerative colitis.

**Table 1:** Patients characteristics pre- and post-intervention, *N* = 1565

Patient Characteristics	Pre-intervention (*n*=632)	Post-intervention (*n*=933)
	% or mean ± SD or median (IQR)	
**Age at admission (months)**	18.8 ± 0.7	18.6 ± 0.6
Under 2 years	67.7	66.6
2-4.9 years	32.3	33.4
**Sex**		
Female	42.2	44.3
**Race**		
Black	51.3	51.2
White	39.6	40.1
Other	9.2	8.7
**Admission type**		
Direct	12.3	13.1
From emergency department	87.7	86.9
**Admission floor**		
A	34.2	35.6
B	37.2	40.0
C	28.6	24.4
**Payment Type**		
Private insurance	24.1	23.5
Medicaid or self-pay	75.9	76.5
**Length of stay in days**	1.9 (2.3)	2.0 (2.4)

Data presented as mean, median, or percent. 2-tailed t test and Chi-square test found no significant differences between groups

*SD* standard deviation, *IQR* Interquartile range

Prior to the intervention, 78.6% of patients were measured for height, compared to 75.8% after the intervention (*p* = 0.19, Table 2). The most common method for measuring height pre-intervention was tape measure (69.2% of patients). Following the intervention, wooden height boards were used for 34.8% of height measurements, while tape measures and wingspan accounted for 42.0% and 3.5% of height measurements, respectively. There were no differences in the proportion of children with height measured or method of height measurement by inpatient floor (data not shown). Digit preference was not common in height measurements (< 15%), and there was no difference in digit preference pre- and post-intervention (Table 2).

**Table 2:** Anthropometry quality indicators, pre- and post-intervention, *N* = 1204

	Pre-intervention		Post-intervention		*P*-value
	n	% or mean± SD	n	% or mean± SD	
**Height measured**					
Yes	497	78.6	707	75.8	0.19
**Measurement method**					
Wooden Height board (Shorr board)	0	0.0	246	34.8	
Tape	344	69.2	297	42.0	<0.01
Wingspan	13	2.6	25	3.5	
Other or missing	140	28.2	139	19.7	
**Plausibility**					
WAZ flag	1	0.2	1	0.2	0.78
HAZ flag	16	3.2	26	3.7	0.67
WHZ flag	12	2.7	4	0.6	<0.01
Height out of range	8	1.8	23	3.5	0.09
**Mean anthropometric indices**					
HAZ, all ages	481	-0.2 ± 1.9	681	-0.5 ± 1.9	0.97
HAZ for age < 2y	331	-0.3 ± 2.1	458	-0.6 ± 2.0	0.70
HAZ for age ≥ 2y	150	-0.1 ± 1.6	223	-0.4 ± 1.7	0.45
HAZ for children measured with board	-	-	238	-0.55 ± 1.8	-
WAZ	451	-0.1 ± 1.5	665	-0.4 ± 1.6	0.17
WHZ	432	0.0 ± 1.60	639	0.0 ± 1.5	0.39
**Digit preference**					
Measurements that would require reclassification	66	13.4	87	12.3	0.62

2-tailed t test and Chi-square test found no significant differences between groups. Extreme z-scores were flagged if implausible as determined by 2006 WHO Growth Standards [6]

*SD* standard deviation, *WAZ* Weight-for-age z-score, *HAZ* height-for-age z-score, *WHZ* weight-for height z-score

One percent of WAZ and 3% of HAZ measurements were flagged as being implausible and there were no differences in WAZ or HAZ flags pre- and post-intervention (Table 2). There was a significant reduction in the number of children flagged for implausible WHZ from 2.7% (12 patients) to 0.6% (4 children) pre- and post-intervention (*p* < 0.01). However, there was a trend toward an increase in height measurements out of range from 1.8% to 3.5% (*p* = 0.09). Following the intervention, height was out of range for 2.0% of patients measured by the wooden boards, compared to 4.0% for tape, 4.0% for wingspan, and 6.5% for other (*p* = 0.18).

**Fig. 2:**
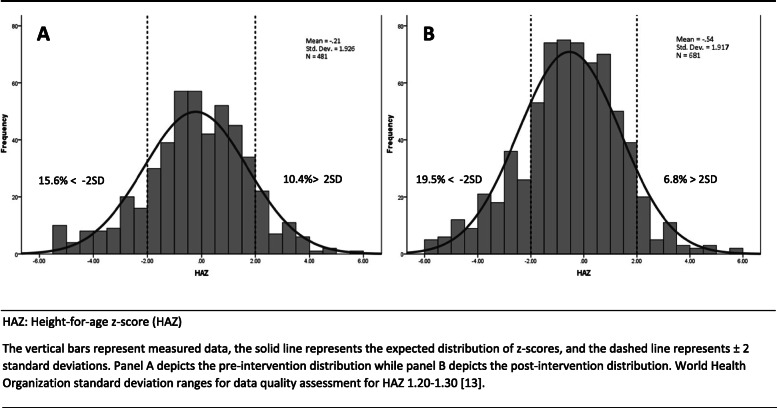
Height-for-age *z*-score distribution for children under 5 years, pre^A^ and post^B^ intervention. HAZ: Height-for-age z-score (HAZ) The vertical bars represent measured data, the solid line represents the expected distribution of z-scores, and the dashed line represents ±2 standard deviations. Panel **a** depicts the pre-intervention distribution while panel **b** depicts the post-intervention distribution. World Health Organization standard deviation ranges for data quality assessment for HAZ 1.20–1.30 [13]

**Fig. 3:**
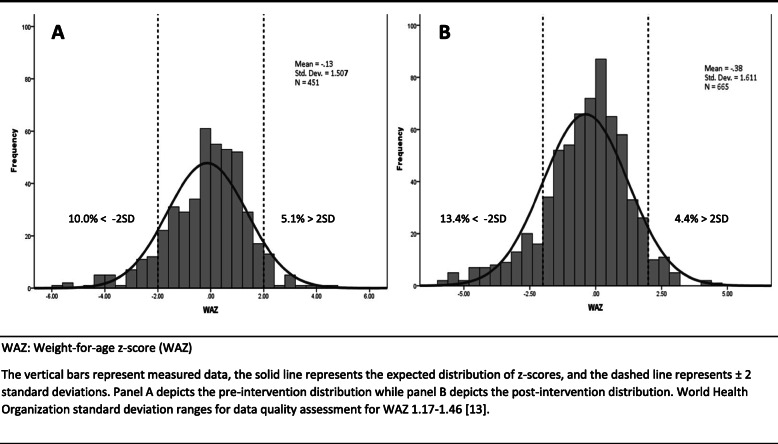
Weight-for-age *z*-score distribution for children under 5 years, pre^A^ and post^B^ intervention. WAZ: Weight-for-age z-score (WAZ) The vertical bars represent measured data, the solid line represents the expected distribution of z-scores, and the dashed line represents ±2 standard deviations. Panel **a** depicts the pre-intervention distribution while panel **b** depicts the post-intervention distribution. World Health Organization standard deviation ranges for data quality assessment for WAZ 1.17–1.46 [13]

**Fig. 4:**
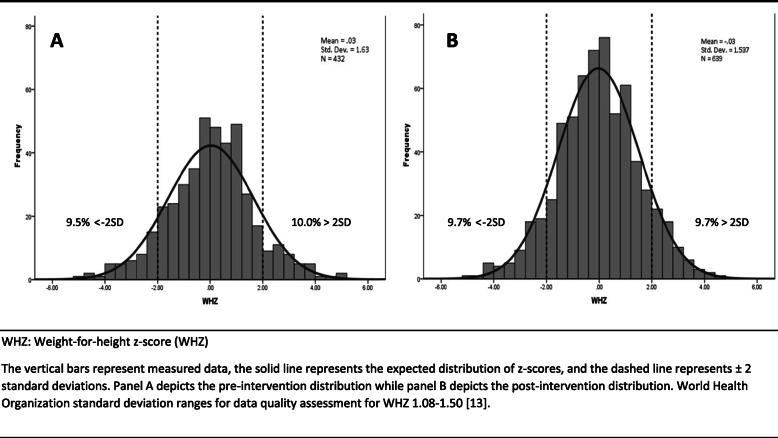
Weight-for-height *z*-score distribution for children under 5 years, pre^A^ and post^B^ intervention. WHZ: Weight-for-height z-score (WHZ) The vertical bars represent measured data, the solid line represents the expected distribution of z-scores, and the dashed line represents ±2 standard deviations. Panel **a** depicts the pre-intervention distribution while panel **b** depicts the post-intervention distribution. World Health Organization standard deviation ranges for data quality assessment for WHZ 1.08–1.50 [13]

Z-score distributions for height-for-age, weight-for-age, and weight-for-height are shown in Figs. 2, 3, and 4, respectively. Prior to the intervention, 5.6%, 10.0%, and 10.0% of participants were stunted, underweight, and wasted, respectively. Following the intervention, 19.5%, 13.4%, and 9.7% of participants were stunted, underweight, and wasted. The SD of HAZ, WAZ, and WHZ pre-intervention were high, 1.9, 1.5, and 1.6, respectively. There were no significant differences between variances pre- and post-intervention across the anthropometric indices. HAZ SD post-intervention appeared slighter greater among children under 2 years (2.0) than children 2 years and older (1.7). The HAZ SD for just the children measured with the wooden board post-intervention (*n* = 246) was 1.8. We found significantly lower variance for both HAZ (*p*-value = 0.004) and WHZ (*p*-value = 0.022) when the wooden board was used post-intervention.

Fifty-three staff participants responded to a questionnaire regarding the quality improvement project. Of these, the majority (71.7%), reported always measuring height of patients following the training; however, only 13.2% reported using the wooden board for height measurements (Table 3). Reasons reported for not routinely measuring height included lack of time, lack of training, and concerns regarding whether the equipment to conduct measurements was adequate. All clinical staff responded that measuring height was important for patient care.

**Table 3:** Staff questionnaire findings

	n (%)
**Did you participate in the anthropometry (ShorrBoard) training?**	
Yes	53 (100)
**Since receiving the training, do you now always measure lengths/heights of your patients?**	
Yes	38 (71.7)
No	15 (28.3)
**Since receiving the training, have you always used the ShorrBoard to measure heights of your patients?**	
Yes	7 (13.2)
No	46 (86.8)
**Why don't you always measure lengths/heights of your patients?**	
I don't have the time	5 (33.3)
I don't think I have the adequate training to measure length/height	1 (6.7)
I don't think we have the proper equipment to measure length/height	1 (6.7)
**Why don’t you always use the ShorrBoard to measure heights of your patients?**	
It is hard to find a second person	31 (67.4)
There is not adequate space for setting up and using the ShorrBoard	20 (43.5)
It takes too long to set up the ShorrBoard	27 (58.7)
The ShorrBoard gets in the way of patient IV's, O2, or other devices	21 (45.7)

Reasons reported for not using the wooden board included taking too much time to set up the board, difficulty finding a second person to assist with measurements, lack of space to conduct measurement, and interference with patients’ intravenous lines, oxygen delivery devices, or other support devices.

Summary of staff attitudes in open-ended questions about the wooden height boards are summarized in Table 4. Staff-reported barriers included the board was heavy, bulky, or cumbersome; the wooden boards were very uncomfortable; the wooden frame was frightening to patients which impacted their participation and cooperation during the measurement; boards often broke during routine use; and boards were shared between patient areas making it difficult to locate the board. Staff emphasized that the availability of wooden height boards on pediatric floors was critical to ensuring implementation of standard methods. If wooden height boards were not readily available, conducting standard anthropometry required additional coordination and communication among clinical staff, which was difficult given their existing responsibilities and busy schedules. Some staff were concerned the wooden boards would contribute to infection transmission. Lastly, standard practice requires the use of two-trained personnel, which staff reported interfered and competed with their individual responsibilities on the floor.

**Table 4:** Summary of staff attitude towards use of wooden height boards

**Traumatizing/Anxiety**	*“[It’s] very traumatizing to place the child into a “wooden Box”. It scares them, makes them cry more, and you can tell the parents are agitated with the process. It's also hard to hold the child down in the box.”*
**Cumbersome**	*“Very heavy and bulky, hard to set up and break down. Must be a easier way to accomplish this.”*
**Unsanitary**	*“Unsanitary, especially when we had to put the board on the crib to measure an infant. If we put it on the floor, we got very strange looks from the parents.”*
**Inaccurate/Difficult to Use**	*“…Also, I feel that as hard as we tried, I don't know whether it was that accurate. The age of the population was also a factor-We get them when they do not feel well, are anxious and have probably been through other procedures (IV's, Labs, Xrays). We all know that heights are important but I did not feel that it was accurate especially when the [patient] was not cooperative. There must be a better way than the ShorrBoard.”*

## Discussion

Although our quality improvement project strived to improve the quality of anthropometric data in pediatric hospitalized patients through staff training and implementation of accurate anthropometry equipment, we did not find improvements in either the frequency of height measurements, nor their plausibility or variance. Additionally, surveyed staff reported that wooden height boards were heavy, cumbersome to assemble, frightening to patients, and required pre-planning and coordination between clinical staff with busy schedules and competing priorities.

Accurate anthropometric measurements are critical to informing clinical care and intervention. Failure to accurately measure anthropometry has been associated with misdiagnosis and treatment of undernutrition and obesity in hospitalized settings [11]. Insufficient training, lack availability of equipment, a lack of incentives provided to clinical staff, difficulty conducting standard anthropometry in clinical settings, and staff non-compliance may explain the lack of improvement in height measurements. The literature supports that staff training is associated with improvement in anthropometric measurements [17]. Future research may consider investigating whether the training provided in this quality improvement project could be revised or built upon; hands-on training sessions, like the Scorpio method [18], or more frequent training sessions may help to ensure accurate measurement [17].

Staff opinions suggest that wooden height boards may not be appropriate for use in pediatric hospitals at this time despite evidence of their superiority [7]. A study by Dixon et al. emphasized the importance of CDC and WHO recommendations to use wooden height boards to improve height measurements but also argued that implementation of standard methodology is not always feasible as a result of environmental obstacles, or participant or staff non-compliance [19]. These factors may also explain our overall null findings.

We found that the variance in height measurements were greater among children under 2 years compared to children 2 years and older, both pre- and post-intervention. This finding is expected as children under 2 years are more difficult to measure and may be less cooperative with staff conducting the measurement. Unfortunately, the majority of children were not measured with the wooden board which may explain our overall null findings. When we examined the variance by measurement method, we found a significantly lower variance for both HAZ (*p*-value = 0.004) and WHZ (*p*-value = 0.022) when the wooden board was used, suggesting that data quality improves when following gold standard protocol. But when taking a closer look at the distribution of HAZ among those children measured with the board, we found that standard deviations continued to exceed WHO data quality cutoffs (SD = 1.8; WHO acceptable range = 1.1–1.3) [13]. These findings may be explained by inadequate training, lack of staff incentives to ensure accurate measurement, or difficulties with conducting gold standard methods in this setting (as supported by our qualitative findings).

Future research may also consider new methods for anthropometric assessment, including the use of 3D imaging technologies. The BINA study examined the use of the AutoAnthro System, a 3D imaging device, and found that the reliability of height measurements was equivalent to gold standard methodology [12]. BINA investigators also found that the technology worked well with cooperative children but additional research is needed to examine the feasibility of 3D imaging technologies among children who have difficulty staying still. One notable improvement of the 3D imaging technology is the reduction in patient burden compared to gold standard methods. Reducing patient burden and stress may help improve staff participation in conducting anthropometric measurements. Another study examined the feasibility of using an adapted laser to perform anthropometric assessment and found that the portable device measured height accurately and reliably [20]. Additionally, the cost of the device was comparable to wooden height boards. Our results suggest these technologies may be viable alternatives to performing anthropometric measurement in field or low-resource settings [21]. It is important to note that while these innovative technologies may provide accurate measurements, initial use and scale up will likely require training, additional cost, and the use of two persons to ensure accurate measurement.

This quality improvement project had multiple strengths. First, our project captured staff reflections and criticisms of conducting standard anthropometry in pediatric hospital settings. These qualitative findings may prove useful in informing strategies to improve accurate anthropometry in pediatric inpatient settings given the structural and practical constraints of the environment. Second, our project collected data on patients with diverse health profiles. Future work may evaluate the impact of outpatient follow-up for malnutrition that is identified during hospital admission, as other studies have shown that in depth discussion and patient education on underweight and overweight status may not be feasible during hospitalization [11].

This project had several limitations. First, training on standard anthropometry was delivered via a computer-based module, and evaluation of the quality of measurement techniques were not performed through a standardization exercise given resource and pediatric staff time constraints. Second, we were also unable to increase the proportion of children who were measured for height. Nearly a quarter of children admitted to the hospital were never measured for height. Of those that were measured for height, nearly a quarter appeared in the electronic data system as “missing or other.” Both of these findings suggest the need for management to ensure implementation of standard protocol when measuring height and during EMR data entry. Third, our quality improvement project focused on undernutrition and did not evaluate overnutrition or obesity, which would be important outcomes to assess in future work. Finally, we were unable to determine the degree to which the variability in our data can be explained by sociodemographic characteristics of our patient population. Further, the dispersion noted in our results may be explained by sociodemographic characteristics of our patient population. Future exploration of this data may consider exploring these associations using scatterplots of the height and weight data, stratified by sociodemographic characteristics like race, gender, and admission type. These exploratory analyses may help elucidate whether there are data clusters of importance, true outliers, evidence of bimodality, other trends and anomalies, as well as allowing visualization of the continuity of the widely dispersed data points.

## Conclusion

Despite recognizing the importance of anthropometric data and implementing standardized training and equipment, pediatric hospital staff had difficulty collecting accurate height measurements. Future research is needed to identify methods to decrease barriers to height measurement to develop more effective approaches to improve the quality of height measurements in hospitalized children.

## Data Availability

The datasets generated and/or analyzed during the current study are not publicly available. These data are property of the Children’s Healthcare of Atlanta but are available from the corresponding author upon reasonable request.

## Abbreviations

WAZ: Weight-for-age z-score
HAZ: Height-for-age z-score
WHZ: Weight-for-height z-score
CDC: Center for Disease Control
WHO: World Health Organization
EMR: Electronic medical record
BMI: Body mass index
SD: Standard deviation
IQR: Interquartile range
3D: Three-dimensional
